# Long Non-Coding RNAs Play a Role in the Pathogenesis of Psoriatic Arthritis by Regulating MicroRNAs and Genes Involved in Inflammation and Metabolic Syndrome

**DOI:** 10.3389/fimmu.2018.01533

**Published:** 2018-07-16

**Authors:** Marzia Dolcino, Andrea Pelosi, Piera Filomena Fiore, Giuseppe Patuzzo, Elisa Tinazzi, Claudio Lunardi, Antonio Puccetti

**Affiliations:** ^1^Department of Medicine, University of Verona, Verona, Italy; ^2^Immunology Area, Pediatric Hospital Bambino Gesù, Rome, Italy; ^3^Department of Experimental Medicine – Section of Histology, University of Genova, Genova, Italy

**Keywords:** psoriatic arthritis, gene expression, long non-coding RNAs, gene module, protein–protein interaction network

## Abstract

Psoriatic arthritis (PsA) is an inflammatory arthritis, characterized by inflammation of entheses and synovium, leading to joint erosions and new bone formation. It affects 10–30% of patients with psoriasis, and has an estimated prevalence of approximately 1%. PsA is considered to be primarily an autoimmune disease, driven by autoreactive T cells directed against autoantigens present in the skin and in the joints. However, an autoinflammatory origin has recently been proposed. Long noncoding RNAs (lncRNAs) are RNAs more than 200 nucleotides in length that do not encode proteins. LncRNAs play important roles in several biological processes, including chromatin remodeling, transcription control, and post-transcriptional processing. Several studies have shown that lncRNAs are expressed in a stage-specific or lineage-specific manner in immune cells that have a role in the development, activation, and effector functions of immune cells. LncRNAs are thought to play a role in several diseases, including autoimmune disorders. Indeed, a few lncRNAs have been identified in systemic lupus erythematosus, rheumatoid arthritis, and psoriasis. Although several high-throughput studies have been performed to identify lncRNAs, their biological and pathological relevance are still unknown, and most transcriptome studies in autoimmune diseases have only assessed protein-coding transcripts. No data are currently available on lncRNAs in PsA. Therefore, by microarray analysis, we have investigated the expression profiles of more than 50,000 human lncRNAs in blood samples from PsA patients and healthy controls using Human Clariom D Affymetrix chips, suitable to detect rare and low-expressing transcripts otherwise unnoticed by common sequencing methodologies. Network analysis identified lncRNAs targeting highly connected genes in the PsA transcriptome. Such genes are involved in molecular pathways crucial for PsA pathogenesis, including immune response, glycolipid metabolism, bone remodeling, type 1 interferon, wingless related integration site, and tumor necrosis factor signaling. Selected lncRNAs were validated by RT-PCR in an expanded cohort of patients. Moreover, modulated genes belonging to meaningful pathways were validated by RT-PCR in PsA PBMCs and/or by ELISA in PsA sera. The findings indicate that lncRNAs are involved in PsA pathogenesis by regulating both microRNAs and genes and open new avenues for the identification of new biomarkers and therapeutical targets.

## Introduction

Psoriatic arthritis (PsA) is a chronic, immune-mediated, asymmetric inflammatory arthritis characterized by inflammation at tendon or ligament insertion sites into bone (enthesitis) and by synovitis, eventually leading to joint erosions and new bone formation (1).

Up to 30% of patients with skin psoriasis may develop PsA and its prevalence is estimated in 1% in the general population. PsA shares genetic and clinical features with other forms of seronegative spondyloarthritis (2, 3). Diagnostic criteria for PsA have not been validated, but the Classification Criteria for PsA (CASPAR criteria), published in 2006, define PsA for the purpose of enrolling patients in clinical trials and provide guidance to clinicians (4, 5). Therefore, the diagnosis of PsA is mainly performed on clinical features after the exclusion of other seronegative arthritides and no diagnostic tests are available so far.

The pathogenesis of PsA is still poorly understood and both autoinflammation and autoimmunity are believed to play a pivotal role in the disease. Synovial tissue in PsA is characterized by T-cell infiltrate, by marked angiogenesis, and by synovial hyperplasia with increased secretion of cytokines and proteases, which may amplify the local inflammatory process eventually leading to joint destruction (6). Tumor necrosis factor-alpha (TNF-α) is a very important inflammatory mediator and has been implicated in the pathogenesis of articular damage in PsA (6). TNF-α inhibitors are currently used in PsA treatment; however, a high percentage of PsA patients does not respond to TNF-α antagonists (1, 7). Therefore, other cytokines have recently become targets of biological agents, such as interleukin-12 (IL-12), interleukin-23 (IL-23), and interleukin-17 (IL-17) (1, 7). Indeed IL-17 plays a fundamental role in disease development and progression (8).

We have reported the findings of the transcriptome analysis in paired synovial tissue and peripheral blood cells of patients with PsA (9). The upregulation of Th-17 cells related genes and of type I interferon (IFN) inducible genes in PsA patients strengthened the hypothesis that PsA has a strong autoimmune origin, since the coactivity of type I IFN and IL-17 pathways is typical of autoimmunity (9). Moreover, we confirmed these findings with a miRNA microarray analysis in PBMCs of PsA patients showing that pathway enrichment analysis on gene targets of deregulated microRNAs (miRNAs) revealed signaling pathways typically implicated in PsA, such as TNF, mitogen-activated protein (MAP) kinase, and wingless related integration site (WNT) cascades (10).

By this study we wanted to provide a more in-depth knowledge on the epigenetic mechanisms that regulate the PsA pathogenesis by analyzing the expression profiles of long non-coding RNAs (lncRNAs) in the same cohort of patients that we studied in our previous work (10). lncRNAs are important molecules that regulate gene expression through multiple mechanisms and are involved in immune and inflammatory pathways (11). As far as we know, no study has yet taken into consideration lncRNAs expression profiles in PsA patients and only a few data have been reported the deregulation of some lncRNAs in psoriasis (12, 13).

Moreover, in this study we offer a sophisticated and integrated analysis of lncRNAs, miRNAs, and gene expression profiles in PsA patients that allows to identify lncRNAs that regulate transcripts effectively modulated in the disease and that are involved in pathogenetically relevant molecular pathways.

## Materials and Methods

### Patients

We studied a cohort of 10 patients (6 males and 4 females, mean age: 53.5 years) affected by PsA, attending the Unit of Autoimmune Diseases, at the University Hospital of Verona, Italy. All patients fulfilled the CASPAR criteria for the diagnosis of PsA: inflammatory musculoskeletal involvement combined with at least three features: (1) evidence of current psoriasis, personal history of psoriasis, and family history of psoriasis in unaffected patients; (2) affected nails (onycholysis and pitting); (3) dactylitis; (4) negative rheumatoid factor (RF); and (5) radiographic evidence of new juxta-articular bone formation (excluding osteophytes) (4). All the patients underwent clinical examination and laboratory evaluation comprehensive of inflammatory markers, such as C-reactive protein and erythrocytes sedimentation rate; RF and anti-cyclic citrullinated peptide antibody detected by ELISA test; antinuclear antibody detected by indirect immunofluorescence on HeLa-derived HEp-2 cells; and genetic screening for the association with the allele HLA-B27. All patients underwent the following instrumental investigations: ultrasonography with Power Doppler to investigate subclinical enthesopathy and synovitis in asymptomatic patients, conventional radiography, magnetic resonance imaging, and scintigraphy. The radiological features of peripheral PsA included asymmetric distribution, participation of distal interphalangeal joints, periostitis, bone density preservation, bone ankylosis, and pencil-in-cup deformity.

The patients were affected by cutaneous or nails psoriasis and were enrolled in the study at diagnosis of peripheral PsA before starting immunosuppressive treatment.

All the participants to the study signed a written informed consent and the local Ethical Committee of the University Hospital of Verona, Verona, Italy, had approved the study protocol. All the investigations have been performed according to the principles of the Helsinki declaration.

### Microarray Analysis

Blood samples were collected in BD Vacutainer K2EDTA tubes using a 21-gauge needle. PBMCs were obtained upon stratification on Lympholyte^®^ cell separation density gradient (Cedarlane, Burlington, ON, Canada). PBMCs composition was similar between patients and controls. Total RNA extraction from PBMCs was performed with miRNeasy mini kit following manufacturer’s protocol (Qiagen GmbH, Hilden, Germany). 500 ng of total RNA were used for sample preparation starting from 5 ml of blood. cRNA preparation, samples hybridization and scanning were performed following the Affymetrix (Affymetrix, Santa Clara, CA, USA) provided protocols, by Cogentech Affymetrix microarray unit (Campus IFOM IEO, Milan, Italy). All samples were hybridized on Human Clariom D (Thermo Fisher Scientific) gene chip and were analyzed using the Transcriptome Analysis Console 4.0 software (Applied Biosystem, Foster City, CA, USA by Thermo Fisher Scientific, Waltham, MA, USA).

The Human Clariom D arrays allow to interrogate more than 540,000 transcripts sourced from the largest number of public databases starting from as little as 100 pg of total RNA.

The Signal Space Transformation-Robust Multi-Array Average algorithm was applied to background-adjust, normalize, and log-transform signals intensity.

Relative gene expression levels of each transcript were validated applying a One-Way analysis of variance (*p* ≤ 0.01) and multiple testing correction. Coding genes and lncRNAs that displayed an expression level at least 1.5-fold different in the test sample versus control sample (*p* ≤ 0.01) were carried forward in the analysis.

The targets (including microRNAs and genes) of all the lncRNAs that satisfied the above-mentioned FC and *p*-value criteria were screened using NPInter v3.0.^1^ This database allows the efficient recovery of all lncRNAs interactions experimentally validated by high-throughput experimental technologies (14, 15).

The list of gene targets of miRNAs that were targeted by significantly modulated lncRNAs was obtained using the FunRich database^2^ (16).

### Protein–Protein Interaction (PPI) Network Construction and Network Clustering

The Search Tool for the Retrieval of Interacting Genes (STRING version 10.5^3^) was used to obtain PPIs pairs that were validated by experimental studies (17) and to construct the PPI networks. Network topological analysis was performed using the Cytoscape software^4^ (18).

High-flow areas (highly connected regions) of the network were detected using the MCODE plugin of Cytoscape, based on the thresholds of *k*-core = 3 and node score cutoff = 0.2.

### Gene Functional Classification and Enrichment Analysis

Genes were functionally classified into canonical biological processes (BPs) on the basis of the gene ontology (GO) annotations.^5^

Biological processes and Pathways enrichment analysis was performed employing FunRich (hypergeometric *p*-value ≤0.05).

### Real-Time PCR

#### lncRNAs Modulated in PsA

For each sample, 500 ng of total RNA was treated with 1 unit of DNase I Amplification Grade (Invitrogen; Carlsbad, CA, USA) according to the manufacturer’s protocol. First-strand cDNA was generated using the SuperScript IV First-Strand Synthesis System (Invitrogen; Carlsbad, CA, USA) with random hexamers, according to the manufacturer’s protocol. Real-time PCR was performed in triplicate with PowerUp™ Sybr^®^ Green reagent (Applied Biosystems; Foster City, CA, USA) in a QuantStudio 6 Flex system (Applied Biosystems; Foster City, CA, USA). Relative expression levels were calculated for each sample after normalization against the geometric mean of the housekeeping genes GAPDH and beta-actin (ACTB) expression. The ΔΔCt method was used for comparing relative fold expression differences. The data are expressed as fold changes with respect to healthy.

#### Genes Modulated in PsA

First-strand cDNA was generated using the SuperScript III First-Strand Synthesis System for RT-PCR Kit (Invitrogen), with random hexamers, according to the manufacturer’s protocol. PCR was performed in a total volume of 25 µl containing 1× Taqman Universal PCR Master mix, no AmpErase UNG, and 2.5 µl of cDNA; pre-designed, gene-specific primers, and probe sets for each gene were obtained from Assay-on-Demande Gene Expression Products (Applied Biosystems).

Real-time PCR reactions were carried out in a two-tube system and in singleplex. The real-time amplifications included 10 min at 95°C (AmpliTaq Gold activation), followed by 40 cycles at 95°C for 15 s and at 60°C for 1 min. Thermocycling and signal detection were performed with 7500 Sequence Detector (Applied Biosystems). Signals were detected according to the manufacturer’s instructions and the relative expression levels were calculated as it has been previously described (9).

### Detection of Soluble Mediators in Sera of PsA Patients

Serum levels of glypican-4, IFN-γ, Wnt-2, mTOR, TNF-α, SPD-1, NFKB p65, NOTCH1, omentin, and adiponectin were detected using commercially available ELISA kits that were supplied by antibodies-online (glypican-4, Wnt-2, and mTOR), LifeSpan BioSciences (TNF-α, NFKB p65, and omentin), IBL International (IFN-γ), R&D (adiponectin), and Ray Biotech (sPD-1).

### Plasmid Construction and Luciferase Reporter Assay

The plasmids to knockdown LINC00909 and LINC00657 were purchased from GenePharma (Shanghai, China).

Human HEK (human embryonic kidney) 293 T cells (1.5 × 10^4^) grown in a 96-well plate were co-transfected with 150 ng of empty vector, 50 ng of firefly luciferase reporter comprising the lncRNAs mut vectors, (Promega, Madison, WI, USA) using Lipofectamie 2000 (Invitrogen, USA). Cells were harvested 48 h after transfection and analyzed using the Dual-Luciferase Reporter Assay System (Promega) according to the manufacturer’s protocol.

## Results

### High-Throughput Long Non-Coding RNA and Gene Expression Profiling in Peripheral Blood Mononuclear Cells of PsA

In order to evaluate the potential role played by lncRNAs in PsA pathogenesis, we performed a gene array study using the Clariom D human gene chip that enabled us to analyze, at the same time, both conventional gene and lncRNA expression profiles.

We compared the lncRNA expression profiles of PBMC samples obtained from 10 PsA patients with 10 PBMC samples obtained from age and sex matched healthy donors and we found that 259 lncRNAs satisfied the Bonferroni-corrected *p* value criterion (*p* ≤ 0.01) and the fold change criterion (FC ≥ |1.5|), displaying robust and statistically significant variation between PsA and healthy controls samples (Table S1 in Supplementary Material). The study was implemented by the analysis of conventional gene expression profiles in the same PsA samples and we found that 1,922 differently expressed genes satisfied the above-mentioned criteria. The complete list of modulated genes can be found in Table S2 in Supplementary Material. In both cases, the arrays were validated by real-time PCR. LncRNAs LUCAT1 and TRIM55-1 were validated by real-time PCR in the entire series of patients analyzed. Significantly different expression levels were found for all tested lncRNAs in PsA as compared to healthy controls (see Figure S1 in Supplementary Material).

**Figure 1:**
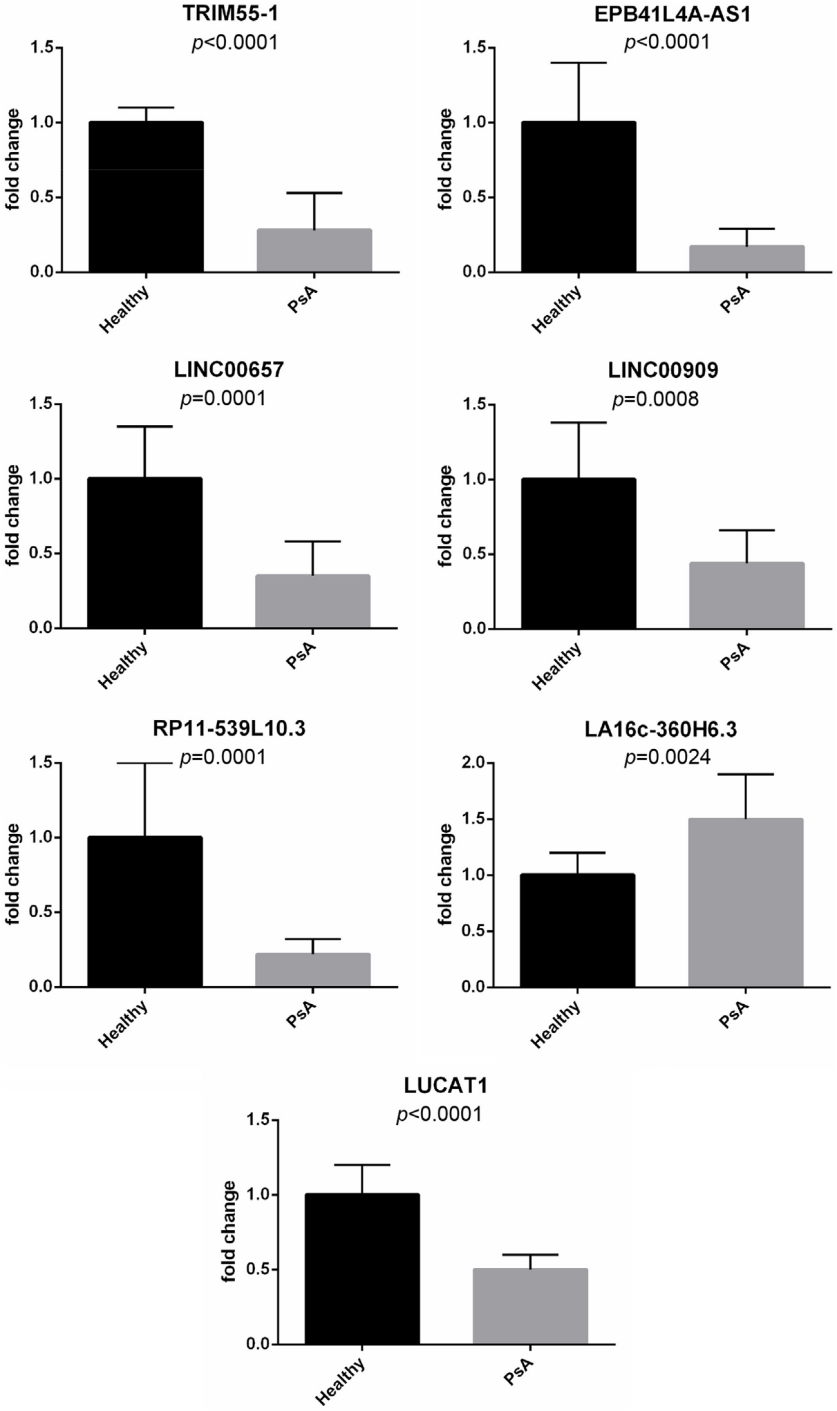
Expression of selected long non-coding RNAs in an expanded panel of psoriatic arthritis patients (20 patients) and healthy controls (20 subjects). Bars indicate SD.

Moreover, real-time PCR analysis for seven lncRNAs was carried out in an expanded panel of PsA patients (20 patients) and healthy controls (20 subjects). A significant modulation of all these lncRNAs was found in all tested patients thus confirming gene array results (see Figure 1).

To gain meaningful insights on the potential role played by modulated lncRNAs in PsA pathogenesis, the complete list of modulated lncRNA was filtered, extracting only those transcripts for which a bona fide target annotations was present in NPInter. By this method 92 lncRNAs were selected (Table S3 in Supplementary Material) and, simultaneously, the list of all gene and microRNA targets of the selected lncRNAs, experimentally validated by high-throughput technologies, was extracted from the same database.

To corroborate our results we narrowed down our analysis to modulate lncRNA that targeted genes that were significantly modulated in the array and miRNAs that we found deregulated in our previous analysis of PsA PBMCs from the same cohort of patients (10) (Table 1). In particular, we found that 15 of these miRNAs modulated in PsA (hsa-miR-130a-3p, hsa-miR-148a-3p, hsa-miR-151a-5p, hsa-miR-17-5p, hsa-miR-186-5p, hsa-miR-199a-3p, hsa-miR-199a-5p, hsa-miR-28-5p, hsa-miR-3135b, hsa-miR-320c, hsa-miR-320d, hsa-miR-331-3p, hsa-miR-423-5p, hsa-miR-451a, and hsa-miR-92a-3p) were targeted by selected lncRNAs. We then extracted from the FunRich database the annotated gene targets of the above-mentioned miRNAs selecting only transcripts that also resulted when modulated in the Clariom D array (Table S4 in Supplementary Material). Table 2 recapitulates the above-selected lncRNAs and targets.

**Table 1:** Selected modulated long non-coding RNAs in psoriatic arthritis patients versus healthy controls.

ID	Fold change	*p*-value	Gene symbol	mRNA accession
TC0500008318.hg.1	−2.37	0.0026	EPB41L4A-AS1	ENST00000413221.2
TC0700007000.hg.1	−2.02	0.0015	HOTAIRM1	ENST00000616712
TC0600008510.hg.1	−2.25	0.0087	KCNQ5-IT1	ENST00000445310
TC0700013567.hg.1	2.05	0.0013	LINC00174	ENST00000416366
TC0700007277.hg.1	2.46	0.0001	LINC00265	ENST00000340510.4
TC1500007707.hg.1	−1.58	0.0089	LINC00593	ENST00000558385.1
TC2000008995.hg.1	−2.27	0.0017	LINC00657	ENST00000565493
TC1800009043.hg.1	−1.84	0.0025	LINC00909	ENST00000577806
TC0200007199.hg.1	2.27	0.0007	LINC00486	ENST00000414054
TC0100009691.hg.1	−1.97	0.0003	RP11-403I13.5	ENST00000443018.1
TC0200010127.hg.1	−3.24	<0.0001	RP11-171I2.4	ENST00000605334.1
TC0200011420.hg.1	−2.68	0.0005	AC133528.2	ENST00000433036.1
TC0400009914.hg.1	−2.37	0.0027	RP11-539L10.3	ENST00000513179.1
TC0500009465.hg.1	−1.74	0.007	RP11-779O18.3	ENST00000523005.1
TC0800007847.hg.1	−14.9	<0.0001	AC084082.3	ENST00000517961.2
TC1100011278.hg.1	1.91	0.0003	RP11-867G23.3	ENST00000501708.1
TC1200006772.hg.1	−1.86	0.0044	RP11-75L1.1	ENST00000541404.1
TC1200010732.hg.1	−3.11	0.0058	RP11-1100L3.8	ENST00000564363.1
TC1400006719.hg.1	−2.54	0.0066	RP11-468E2.5	ENST00000558478.1
TC1400009275.hg.1	−1.61	0.0027	RP11-930O11.2	ENST00000560296.1
TC1600009188.hg.1	2.21	0.0021	LA16c-360H6.3	ENST00000574245.1
TC1700007241.hg.1	2.64	0.0008	RP11-283C24.1	ENST00000578585.1
TC2100007843.hg.1	−1.78	0.0016	AF131217.1	ENST00000430247.1
TC2200008462.hg.1	−3.38	0.0058	RP3-430N8.10	ENST00000602955.1
TC1500010312.hg.1	−2.52	0.0064	RP11-815J21.2	ENST00000561409.1
TC1800007426.hg.1	−3.49	0.0032	RP11-1151B14.4	ENST00000591360.1
TC1900011833.hg.1	1.81	0.0031	CTB-25B13.12	ENST00000588225.1
TC1900007159.hg.1	−1.77	0.0084	CTB-55O6.10	ENST00000590715.1
TC1200008393.hg.1	−1.74	0.0028	RP11-981P6.1	ENST00000552778.1
TC1200008425.hg.1	−2.1	0.0024	RP11-796E2.4	ENST00000499685.2
TC1400009962.hg.1	−2.61	0.0006	RP11-471B22.2	ENST00000555853.1
TC1600006833.hg.1	2.16	0.0024	RP11-77H9.5	ENST00000564919.1
TC1400009667.hg.1	1.88	0.0092	RP4-693M11.3	ENST00000557304.1
TC1000009009.hg.1	−1.6	0.0098	RP11-498B4.5	ENST00000433600.1
TC1400010386.hg.1	1.71	0.0064	CTD-3051D23.4	ENST00000553344.2
TC1200008527.hg.1	−2.91	0.0065	RP11-256L6.3	ENST00000551849.1
TC0200007485.hg.1	1.61	0.0048	AC016722.4	ENST00000429761.1
TC1400007302.hg.1	1.95	0.0041	CTD-2002H8.2	ENST00000557322.1
TC0500008150.hg.1	−2.4	0.0066	CTD-2260A17.1	ENST00000512856.1
TC0600010636.hg.1	−1.89	0.0005	RP3-406P24.3	ENST00000415144.1

**Table 2:** Long non-coding RNAs (LncRNAs) and their targets modulated in psoriatic arthritis.

LncRNAs	Gene targets	miRNA targets
LINC00174	CPSF7	
LINC00265	IGF2BP2	
LINC00593	UPF1	
LINC00657	CPSF7, FXR1, HNRNPC, NUDT21 UPF1, ZC3H7B	hsa-miR-130a-3p
		hsa-miR-17-5p
		hsa-miR-186-5p
		hsa-miR-199a-3p
		hsa-miR-199a-5p
		hsa-miR-28-5p
		hsa-miR-320c
		hsa-miR-320d
		hsa-miR-331-3p
		hsa-miR-423-5p
		hsa-miR-451a
LINC00909	UPF1	hsa-miR-130a-3p
		hsa-miR-148a-3p
		hsa-miR-28-5p
		hsa-miR-320c
		hsa-miR-320d
LINC00486	FXR1, UPF1	
EPB41L4A-AS1	CPSF7, UPF1	hsa-miR-130a-3p
		hsa-miR-17-5p
HOTAIRM1	CPSF7, UPF1	
KCNQ5-IT1	CPSF7, UPF1	
RP11-403I13.5		hsa-miR-92a-3p
RP11-171I2.4	CPSF7	
AC133528.2	CPSF7	
RP11-539L10.3		hsa-miR-148a-3p
		hsa-miR-199a-3p
RP11-779O18.3	HNRNPC, UPF1, ZC3H7B	
AC084082.3	CPSF7, NUDT21, UPF1	hsa-miR-331-3p
RP11-867G23.3	UPF1, IGF2BP2	
RP11-75L1.1	UPF1	
RP11-1100L3.8	UPF1	hsa-miR-17-5p
		hsa-miR-423-5p
RP11-468E2.5	UPF1	
RP11-930O11.2	UPF1	
LA16c-360H6.3	IGF2BP2	hsa-miR-17-5p
RP11-283C24.1	UPF1	
AF131217.1	UPF1	
RP3-430N8.10		hsa-miR-331-3p
RP11-815J21.2		hsa-miR-186-5p
RP11-1151B14.4	UPF1	
CTB-25B13.12	UPF1	
CTB-55O6.10	CPSF7, UPF1	
RP11-981P6.1	CPSF7, UPF1, ZC3H7B	
RP11-796E2.4	UPF1	hsa-miR-3135b
RP11-471B22.2	CPSF7, IGF2BP2, UPF1, ZC3H7B	
RP11-77H9.5	CPSF7, HNRNPC, IGF2BP2, UPF1	
RP4-693M11.3	UPF1	
RP11-498B4.5	UPF1	
CTD-3051D23.4	IGF2BP2	
RP11-256L6.3	NUDT21, UPF1	
AC016722.4	CPSF7, UPF1, ZC3H7B	
CTD-2002H8.2	CPSF7, HNRNPC, NUDT21, UPF1, ZC3H7B	
CTD-2260A17.1		hsa-miR-151a-5p
RP3-406P24.3	CPSF7	

In summary, we selected only those lncRNAs, that targeted miRNAs and genes with evidence of modulation in our PsA samples to trace, with a good confidence, lncRNAs–miRNAs–genes interactions that are expected to be established in the course of PsA.

To validate our results we conducted a LINC00909siRNA and a LINC00657siRNA silencing in human 293 T cells, to explore whether this knockdown altered the expression levels of selected miRNAs targeted by LINC00909 (miR-148a-3p and mir-28-5p) and by LINC00657 (miR-130a-3p and miR-17-5p). We observed that the silencing of the two lncRNAs significantly increased the level of expression of their targeted miRNAs.

The registered percentages of increase were: 70 ± 3.8 and 78 ± 1.4% for miR-148a-3p and mir-28-5p, respectively, and 75 ± 2.3 and 80 ± 1.53% for miR-130a-3p and miR-17-5p, respectively.

### PPI Network of Modulated lncRNAs in PsA

The lncRNA expression profiling of PsA PBMCs was integrated with a network analysis. We, therefore, inspected, by a bioinformatic methodology, all the functional and experimentally validated interactions among the protein products of genes targeted by lncRNAs and by the 15 above-mentioned miRNAs (230 genes) that were selected as previously described. Then, taking in account of these interactions, we constructed a PPI network which showed a good PPI enrichment *p*-value (0.00041). In the obtained network 229 of the above-mentioned genes (nodes), were connected by 195 pairs of interactions (edges). Since 229 out of 230 of lncRNAs and miRNAs gene targets that we used as input for the network analysis, resulted connected in the PPI network, we could observe that the selected lncRNAs may act in an integrated fashion in the disease.

**Figure 2:**
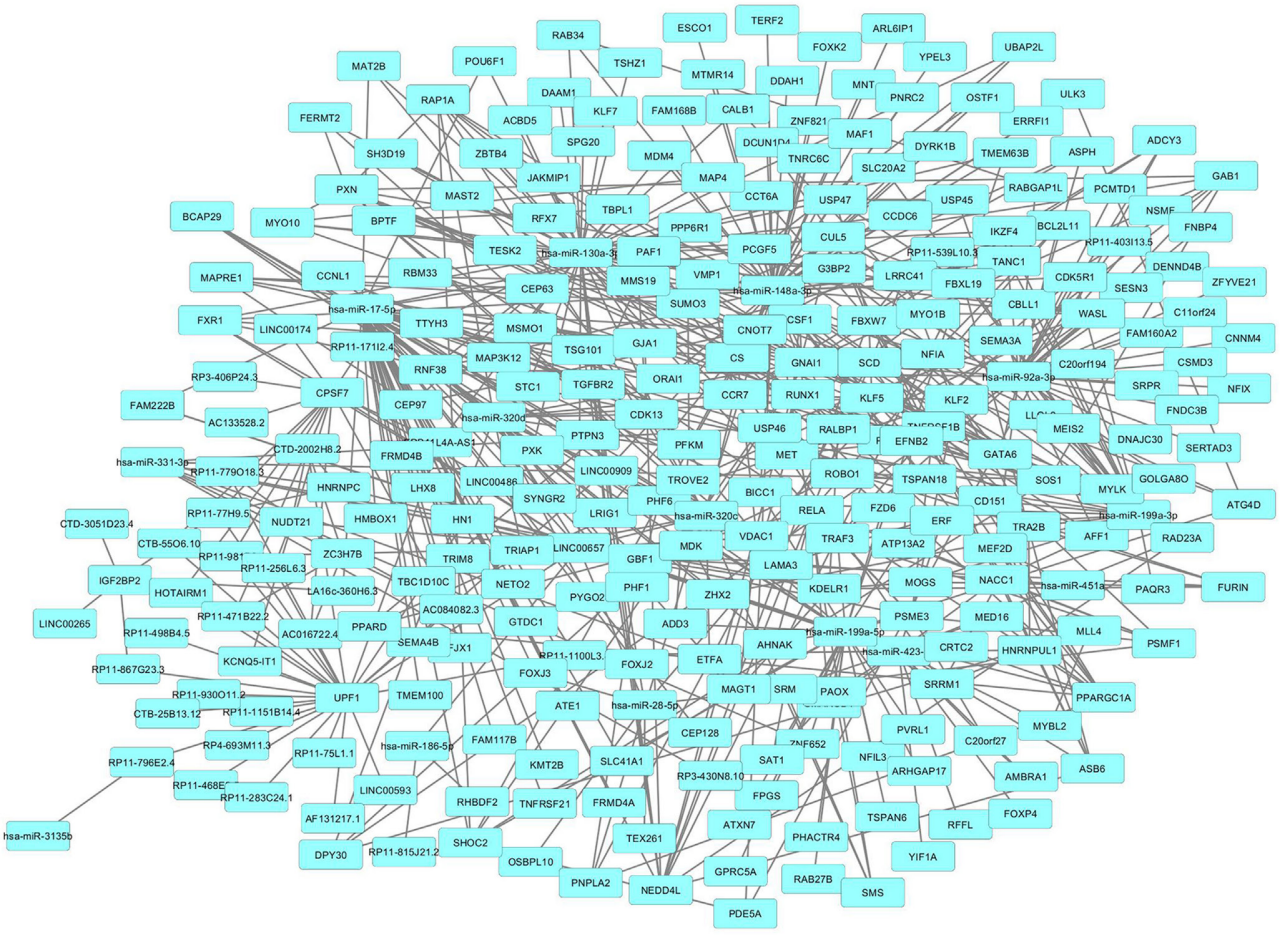
Protein–protein interaction (PPI)-network of differently expressed genes in psoriatic arthritis (PsA) targeted by the selected long non-coding RNAs and microRNAs modulated in PsA.

**Figure 3:**
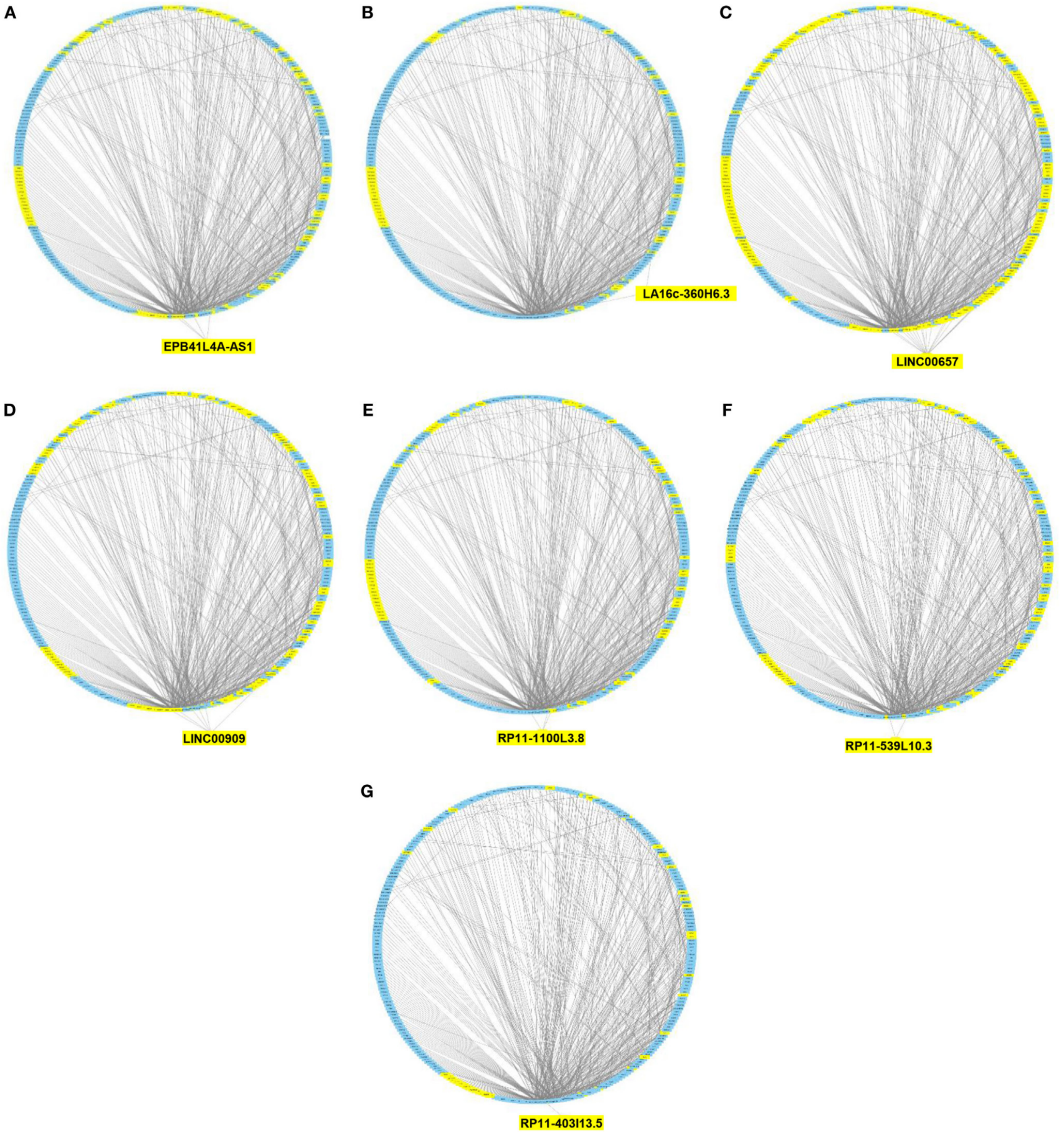
Protein–protein interaction (PPI) network of differently expressed genes targeted by selected microRNAs and long non-coding RNAs (lncRNAs). Graphical representation of the connections among genes in the PPI network and EPB41L4A-AS1 **(A)**, LA16c-360H6.3 **(B)**, LINC00657 **(C)**, LINC00909 **(D)**, RP11-1100L3.8 **(E)**, RP11-539L10.3 **(F)**, and RP11-403I13.5 **(G)**. In the PPI network all genes are ordered around a circle based on their degree of connectivity (i.e., number of edges). Genes directly or indirectly targeted by the lncRNAs are highlighted in yellow.

We then imported in Cytoscape the PPI network, adding to its scaffold the lncRNAs-genes, miRNAs-genes, and lncRNAs–miRNAs interactions that we selected as we explained above. Thus, we obtained an implemented network that is showed in Figure 2. A topological analysis of this network was performed to highlight lncRNAs that were highly connected to the PPI network genes (i.e., targeting a large number of PPI-network genes) also considering all the lncRNA–gene interactions mediated by the selected miRNAs (i.e., lncRNA–miRNA–gene interactions). By this approach we could select seven lncRNAs, including EPB41L4A-AS1, LA16c-360H6.3, LINC00657, LINC00909, RP11-1100L3.8, RP11-539L10.3, and RP11-403I13.5 that displayed the highest connectivity in the network. As shown in Figures 3A–G (where genes of the PPI network are ordered around a circle based on their degree of connectivity, i.e., number of edges) the above-mentioned lncRNAs directly or indirectly targeted genes that, in most cases, were distributed in areas of the network characterized by a high degree of connectivity. This observation indicated that, since these lncRNAs targeted highly interconnected genes, they may have a broad effect in the disease that goes beyond the modulation of single target gene(s).

### Modular Analysis of Genes and lncRNAs Modulated in PsA

**Figure 4:**
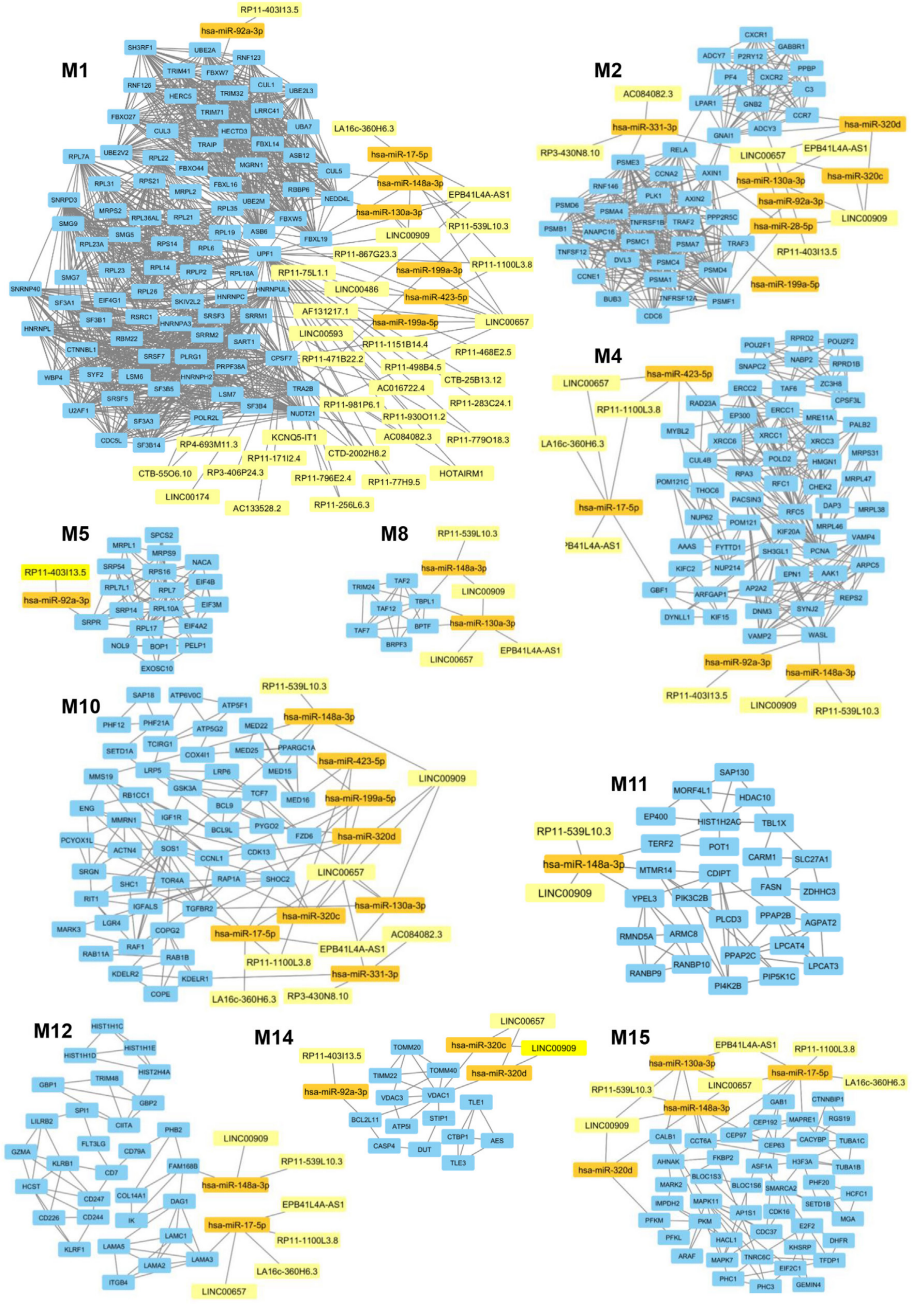
Gene modules that are targeted by the selected long non-coding RNAs (lncRNAs) modulated in psoriatic arthritis. In each module (M) genes are represented by light blue rectangles whereas targeting microRNAs and lncRNAs are represented by orange and yellow rectangles, respectively.

In a second part of our analysis we wanted to dissect the global impact of lncRNAs on the entire PsA transcriptome (i.e., on the 1,922 modulated genes in PsA). Since the targeting of genes with more interacting partners may have a broader impact on the global transciptome than the modulation of few isolated genes, we aimed to inspect if the modulated lncRNAs may display connections with highly interacting genes in PsA. To this purpose, we built the PPI network that included the protein products of all modulated genes in PsA that showed experimentally validated interactions obtaining a wide network containing 1,637 nodes and 9,415 edges and showing a very good PPI enrichment *p*-value (*p* < 10–16). Then we performed a modular analysis to find areas of the network that included the most highly connected genes (modules). By this analysis we could extract 16 modules (M1–M16, see Table S5 in Supplementary Material). All the 16 modules were imported in Cytoscape along with all the previously selected interactions with miRNAs and modulated lncRNAs that we also considered in the network analysis described in the previous paragraph. The graphical visualization of genes connected in modules and their interactions with lncRNAs showed that the majority of the 16 modules (10/16) were directly or indirectly targeted by modulated lncRNAs, including M1, M2, M4, M5, M8, M10, M11, M12, M14, and M15 (Figure 4). Moreover, we could observe that the vast majority of modules was targeted by the above-mentioned lncRNAs that included LINC00909 (targeting 9/10 modules) LINC00657 (targeting 8/10 modules), EPB41L4A-AS1, RP11-539L10.3 (targeting 7/10 modules), LA16c-360H6.3, RP11-403I13.5, and RP11-1100L3.8 (targeting 5/10 modules) (Table S6 in Supplementary Material).

### Functional Analysis of the Targeted Modules and Their Targeting lncRNAs

**Figure 5:**
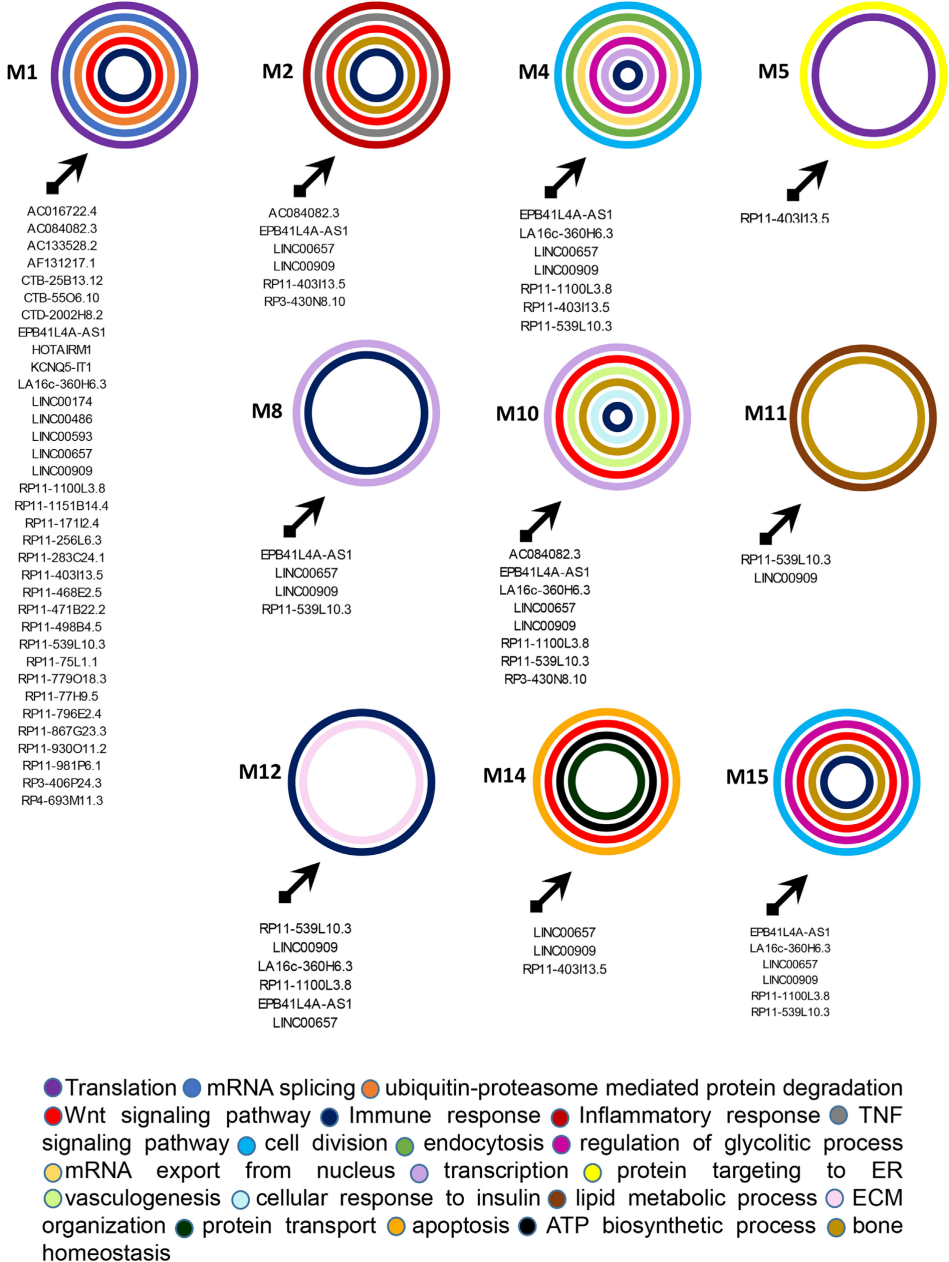
Functional classification of genes included in the targeted modules. Graphical representation of long non-coding RNAs targeting the gene modules and of the most relevant biological processes (BPs) in which genes in each module can be classified. The BPs are represented by colored circles (each color corresponds to a different BP). The size of circles does not correspond to the number of genes that are classified in each BP.

To dissect the possible role played by the module-targeting-lncRNAs we performed a functional classification of genes included in the 10 targeted modules, based on the GO classification criteria. Figure 5 recapitulates all the most relevant GO BPs in which the genes of targeted modules are classified.

M1 was the most targeted module and 35 lncRNAs showed direct or indirect connections with it (see Table S6 in Supplementary Material). The 88 genes included in this module were mostly related to translation, mRNA splicing, and protein ubiquitination GO BPs. Among genes included in protein ubiquitination, nine genes belonged to the “proteasome-mediated ubiquitin-dependent protein catabolic process” BP, including CUL1, CUL3, FBXW5, FBXW7, FBXL19, HECTD3, NEDD4L, RNF126, and UBE2A. Interestingly, it has been recently observed that ubiquitin–proteasome-mediated protein degradation plays a role in modulating the balance between bone formation and bone resorption, since it is involved in signaling pathways that are crucial for bone homeostasis like RANK/NF-κB pathway and the Wnt/β-catenin pathway (19). In addition CUL3 is also associated with the “Wnt signaling pathway” BP. 12 genes were targeted in the M1, including CUL5, TRA2B, SRRM1, ASB6, HNRNPUL1, CPSF7, HNRNPC, NUDT21, UPF1, and the above-mentioned FBXL19, NEDD4L, and FBXW7. UPF1 was the most targeted gene in M1. It is critical for the activity of Regnase-1 also called MCPIP1 (monocyte chemotactic protein-1-induced protein-1), a crucial molecule that regulates immune response avoiding over-activation of the immune system (20). Interestingly, the gene FBXL19 represents a susceptibility locus for PsA (21). Finally, other two genes of M1, TRIM32, and HERC5 play a role in the innate immune response and, in particular, HERC5 activates IRF3 (22).

M2 was targeted by 6 lncRNAs and included 40 genes that were mainly involved in cell division, inflammatory response (i.e., CCR7, CXCR1, CXCR2, PF4, and PPBP/CXCL7), tumor necrosis factor (TNF)-mediated signaling pathway (i.e., TNFRSF1B, TNFSF12, TNFRSF12A, and PSMA7), and Wnt signaling pathway (i.e., AXIN1, DVL3, GNB2, PPP2R5C, PSMA4, PSMB1, PSMC4, PSMD4, and RNF146) BPs. Interestingly, the expression of CXCL7 is increased in Rheumatoid arthritis (AR) synovia (23). Moreover AXIN2 another gene included in M2 is related to the intramembranous ossification BP. Eight genes were targeted in M2, including ADCY3, GNAI1, PSME3, PSMF1, RELA, TRAF3, and the above-mentioned TNFRSF1B and CCR7. In particular, the expression of CCR7 is stimulated by TNF-α and interleukin 1-beta in osteoclasts (24). Moreover, RELA is involved in peripheral regulatory T cell-induced tolerance (25) and TRAF3 is required for T cell and invariant natural killer T cell effector functions (26). In addition, TRAF3 is also involved in limiting osteoclastogenesis, indeed RANKL-induced degradation of TRAF3 enhances TNF-induced osteoclastogenesis (27).

M4 was targeted by seven lncRNAs and included 57 genes that were mainly involved in cell division, DNA repair, endocytosis, mitochondrial translation, mRNA export from nucleus, regulation of glycolytic process, and transcription. In addition, this module included two genes (i.e., MRE11A and XRCC6) associated with the BP “positive regulation of type I interferon production” and the gene POU2F2/OCT2 that increases the differentiation of antibodies secreting activated B cells (28). In this module the genes GBF1, MYBL2, RAD23A, and WASL were targeted.

M5 was targeted by the RP11-403I13.5 lncRNA and included 19 genes. Protein targeting to ER and translation were the most represented BPs in this module.

M8 was targeted by four lncRNAs (EPB41L4A-AS1, LINC00657, LINC00909, and RP11-539L10.3) and in this module seven genes were connected, including BRPF3, TAF12, TAF2, TAF7, TBPL1, TRIM24, and BPTF almost associated with the “translation” BP. Among these, BPTF is important for the homeostasis of T cells and is crucial for the maintenance and function of regulatory T (Treg) cells (29).

M10 was targeted by 8 lncRNAs and included 49 genes. These genes were associated with many BPs, including cell proliferation, signal transduction, transcription, Wnt signaling pathway (i.e., BCL9L, FZD6, LRP6, ATP6V0C, BCL9, and PYGO2), vasculogenesis (i.e., ENG and TGFBR2), bone resorption (i.e., TCIRG1), negative regulation of bone mineralization (i.e., SRGN), osteoblast differentiation (i.e., LGR4), and osteoblast development (i.e., LRP5). Several genes were associated with BPs related to glucose homeostasis like, for example, PPARGC1A (cellular glucose homeostasis), GSK3A (cellular response to insulin), IGF1R (insulin receptor signaling pathway), RAF1 (insulin secretion), and RAP1A (positive regulation of glucose import). In addition other genes were classified into BPs related to adaptive immune response like T cell differentiation (i.e., RIT1 and TCF7), and T cell cytokine production (KDELR1) and B cell homeostasis (SOS1). In M10 12 genes were targeted, including SHOC2, CCNL1, CDK13, MED16, MMS19, and the above-mentioned FZD6, PYGO2, KDELR1, PPARGC1A, RAP1A, SOS1, and TGFBR2.

M11 was targeted by 2 lncRNAs and included 28 genes. A large number of these genes were assigned to “lipid metabolic process” (i.e., PLCD3, PPAP2B, SLC27A1, and TBL1X), “phospholipid metabolic process” (i.e., AGPAT2, LPCAT3, LPCAT4, and PPAP2C), and to “phosphatidylinositol biosynthetic process” (CDIPT, MTMR14, PI4K2B, and PIK3C2B) BPs. Interestingly, two genes namely CARM1 and FASN were associated with “endochondral bone morphogenesis” and “osteoblast differentiation” BPs, respectively. In this module, TERF2, YPEL3, and the above-mentioned MTMR14 were targeted.

M12 was targeted by 6 lncRNAs and included 30 genes. We observed that several genes of this module classified in BPs related to the immune response, including SPI1/PU.1 (lymphoid progenitor cell differentiation), CD244 and KLRF1 (innate immune response), CD226 (positive regulation of natural killer cell cytokine production), CD7 (T cell activation), CD247 (T cell receptor signaling pathway), GBP1 (regulation of T cell receptor signaling pathway), CD79A (B cell activation), GBP1, GBP2, and CIITA (type I interferon signaling pathway), HCST, LILRB2, and KLRB1 (regulation of immune response), GZMA (immune response) and SPI1/PU.1 (lymphoid progenitor cell differentiation), and CIITA (positive regulation of MHC class I and of MHC class II biosynthetic process). In particular, the transcription factor SPI1/PU.1 is crucial for the development of interleukin-9-producing helper T cells (Th9) (30, 31) that have been recently involved in the PsA pathogenesis (32).

Moreover five genes, including DAG1, COL14A1, LAMA2, LAMA3, and LAMC1 were associated with the “extracellular matrix organization” BP. LAMA3 and FAM168B were targeted in this module.

M14 was targeted by three lncRNAs and included 14 genes that were mainly associated with protein targeting to mitochondrion (TOMM20 and TOMM40), protein import into mitochondrial inner membrane (TIMM22), ATP biosynthetic process (ATP5I), apoptosis (BCL2L11 and CASP4), and in Wnt signaling pathway (TLE1 and TLE3) BPs. In this module the genes BCL2L11 and VDAC1 were targeted.

Finally, M15 was targeted by 6 lncRNAs and included 44 genes. In this module, we observed the presence of genes classified in several BPS, including cell division (TUBA1C, TFDP1, CEP192, CEP63, and MAPRE1) glycolytic process (PFKL, PFKM, and PKM), Wnt signaling pathway (MARK2, CTNNBIP1, EIF2C1, and TNRC6C), osteoblast differentiation (ASF1A and MAPK11), and regulation of type I interferon-mediated signaling pathway (CDC37). Genes targeted in this module were: AHNAK, CALB1, CCT6A, CEP63, CEP97, GAB1, MAPRE1, PFKM, and TNRC6C.

CALB1 plays a role in differentiation and matrix formation in osteoblasts (33), GAB1 is important for normal postnatal bone homeostasis (34) and MAPRE1 regulates cell–cell adhesion-induced osteoblast differentiation (35).

Since disease can be viewed as the result of perturbations of complex signaling networks, a pathway enrichment analysis was performed, in each module, using FUnrich. This analysis showed that the most represented enriched pathways were related to immune response (including signaling of both adaptive and innate immune response, type I interferon and gamma interferon signaling), bone homeostasis, metabolism, and cell adhesion.

**Figure 6:**
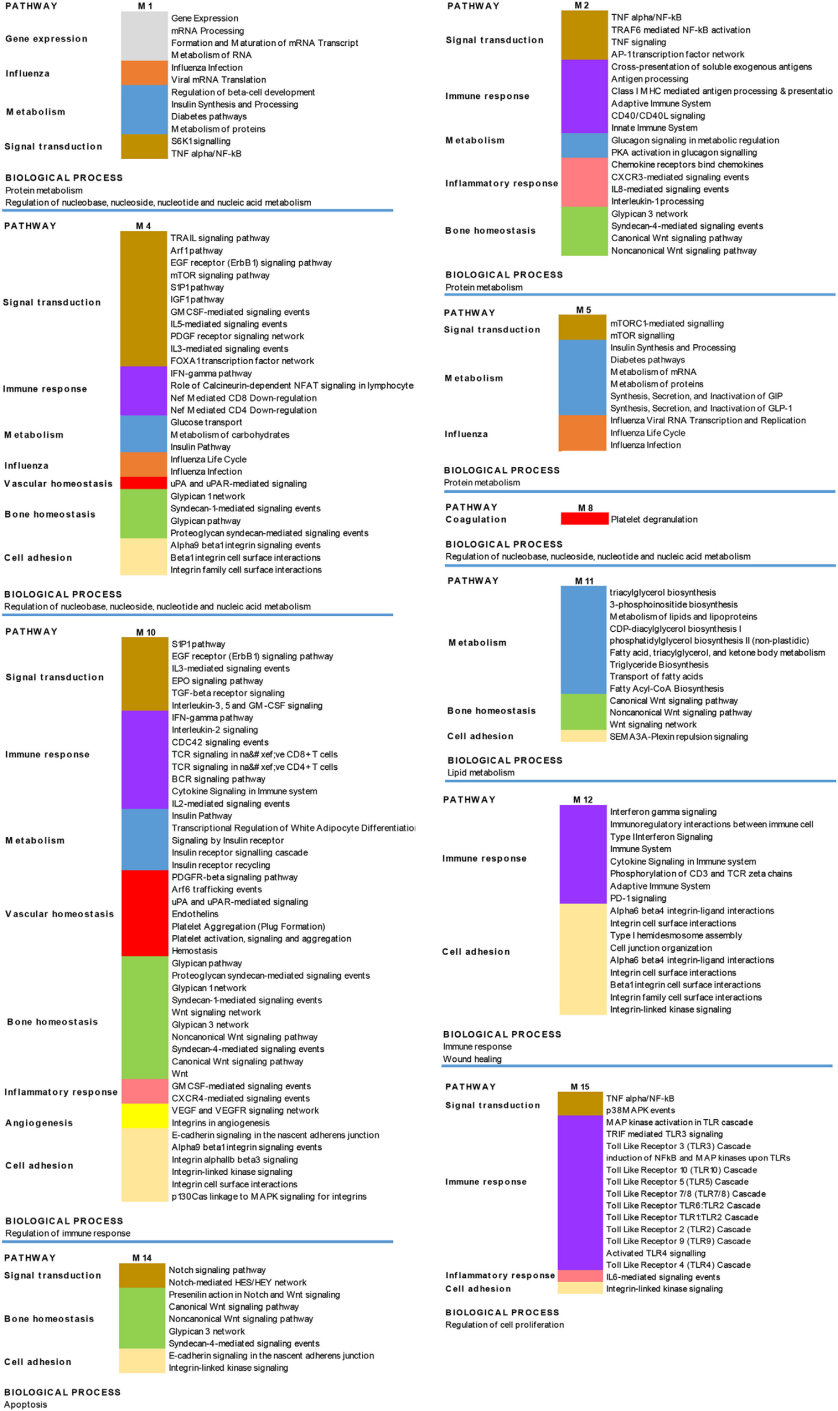
Biological processes and pathways enrichment analysis of the targeted modules. Selection of the different enriched pathways (*p* < 0.05) in the 10 targeted modules (M). Pathways that are enriched in more than one module are labeled by the same color in the different modules.

The results obtained are shown in Figure 6, which shows a schematic representation of the most relevant enriched signaling pathways (*p* ≤ 0.05).

To provide experimental evidence that pathways identified by the throughput analysis of modulated genes and lncRNAs in PsA are active in the disease, we performed a two step validation of the presented results. Indeed, first we validated by real-time PCR the modulation of genes ascribed to several signaling pathways, including SH3GL1, EPN1, and NUP214 (mTOR signaling), TRAF2, TNFRSF1B, and RELA (TNF/NF-κB signaling), MAPK7 and MAPK11 (toll-like receptors signaling), TCF7, BCL9, and LRP6 (Wnt signaling), MED15, ENG, and ACTN4 (glypican signaling), GSK3A, IGF1R, and PARGC1A (insulin signaling), and CIITA IFI27 and MYD88 (type I interferon signaling). All the tested transcripts were overexpressed in PsA patients when compared to healthy subjects (see Figure S2 in Supplementary Material).

**Figure 7:**
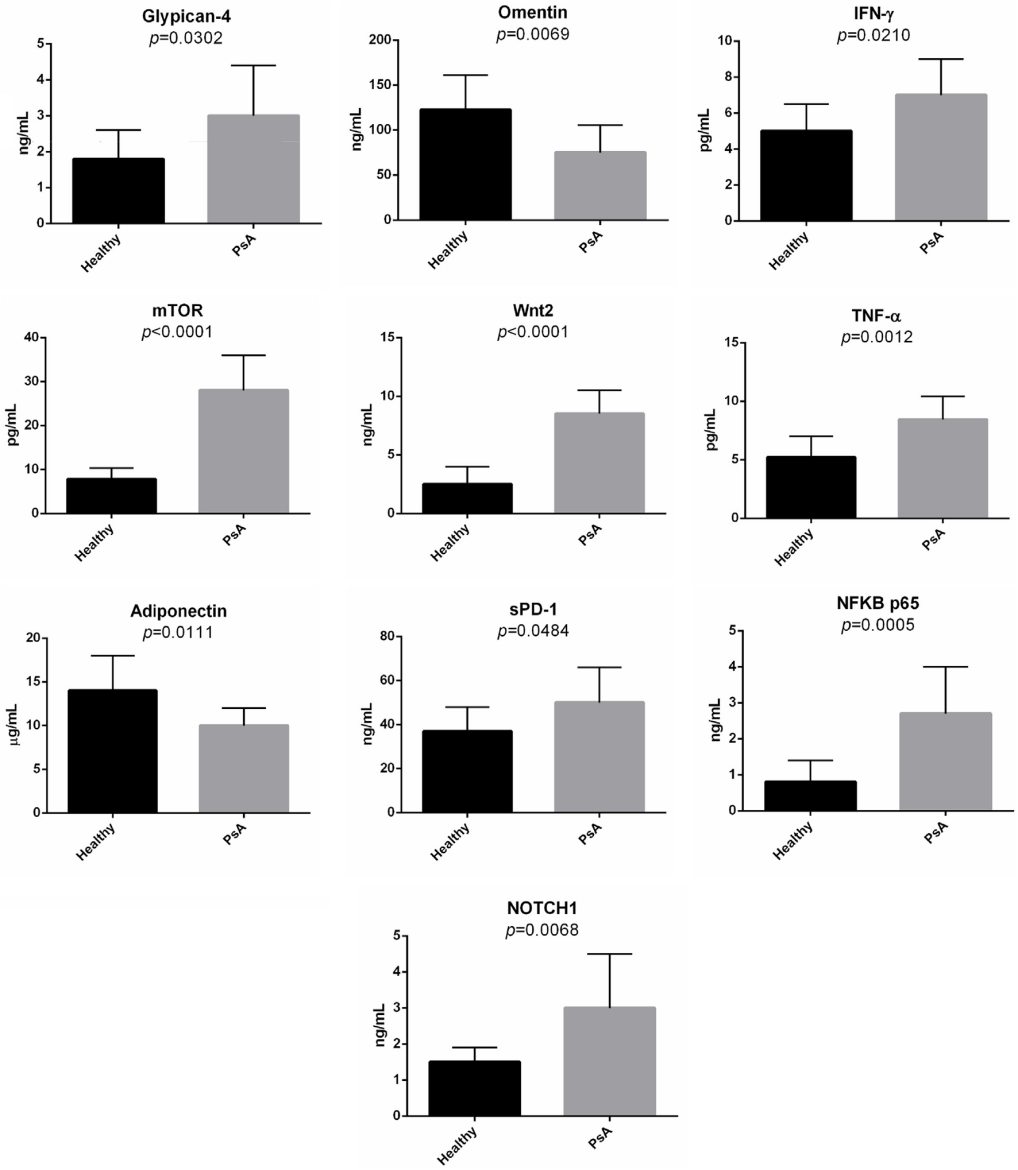
Serum levels of selected molecules in psoriatic arthritis (PsA) patients and in normal subjects. The histograms represent the mean of the results obtained in 20 healthy donors and in 20 PsA patients.

Second, in the sera of patients with PsA, we evaluated the presence of several molecules associated with the pathways modulated in PsA. Indeed, we decided to quantify the serum levels of glypican-4, IFN-γ, Wnt-2, mTOR, TNF-α, sPD-1, NFKB p65, NOTCH1, omentin, and adiponectin. Figure 7 shows the concentration of these molecules in the sera of the PsA patients. The serum levels of all the tested molecules were significantly increased in PsA patients when compared to healthy donors with the exception of adiponectin and omentin that were significantly decreased in PsA sera.

## Discussion

Psoriatic arthritis is a chronic inflammatory arthritis that affects 10–30% of patients with skin psoriasis. IL-17, TNF-α, and type I IFNs play a fundamental role in the pathogenesis of the disease and monoclonal antibodies targeting IL-17 and TNF-α along with IL-12/IL-23 are the main biotechnological treatments that have a dramatic good response in PsA and in skin psoriasis. As we have previously reported (9), the involvement of the mentioned cytokines is typical of an autoimmune process and support the hypothesis of the autoimmune origin of the disease. In particular, IL-17 is able to synergize with TNF-α, IL-22, and other cytokines, including IL-6 and IL-8, in sustaining the inflammatory process at different sites and in favoring the development of comorbidities that are typical of psoriasis, such as PsA, metabolic syndrome, obesity, and cardiovascular disease.

Long non-coding RNAs control gene expression at multiple levels, including epigenetic regulation, chromatin remodeling, and post-transcriptional gene regulation. Accumulating evidences indicate that lncRNAs can be involved in different types of immune-mediated human diseases, including autoimmune diseases (11). The role played by lncRNAs in PSA pathogenesis has not been elucidated yet and a comprehensive analysis of lncRNA expression profiles in PsA has not been performed. We, therefore, aimed to identify lncRNA expression signatures associated with PsA, analyzing the expression of a vast array of lncRNA in PBMC cells from PsA patients by microarray analysis.

The applied criteria for the selection of modulated lncRNAs included a multiple step process that combined the lncRNAs expression analysis to the conventional gene expression and miRNAs profiling in the same cohort of patients.

By this approach we were able to select deregulated lncRNAs that were as much as possible correlated to the PsA transcriptome.

Through a complex network analysis of the whole set of molecular interactions among modulated genes and lncRNAs we further wanted to select those lncRNAs that targeted modules of most highly connected genes in the PsA interactome, since they may have a major impact on PsA gene modulation. That, for example, has led us to favor lncRNAs with high connectivity rather than poorly connected lncRNAs (even) with higher fold change. The functional analysis of the above-mentioned highly connected modules revealed that the modulated lncRNAs targeted genes involved in BPs that play a pivotal role in the PsA pathogenesis such as, for example, immune response (including B and T cell activation) inflammatory response, TNF, Wnt and type I interferon-mediated signaling, bone resorption, bone mineralization, and glyco-lipid metabolic process (see Results), thus indicating that the selected lncRNAs regulated the three major aspects of the disease, including skin involvement, osteo-articular features, and metabolic syndrome.

As it has recently acquired in molecular biology, diseases are now viewed in the context of signaling pathways perturbation. We, therefore, performed a pathway enrichment analysis of all targeted gene modules and, by this approach we reached a non-ambiguous identification of important pathways that can be modulated by the selected lncRNAs. Our analysis confirmed that these lncRNAs play a role in the regulation of signaling pathways associated with both adaptive and immune response and with autoimmune diseases (i.e., type I interferon signaling pathway). In addition, they regulate important signaling involved in bone homeostasis including glypican, syndecan, and Wnt pathways.

Moreover, these results revealed the presence in PsA of lncRNAs that modulate the signaling pathway associated with influenza, thus highlighting the possible role played by influenza viruses in triggering PsA and suggesting the possible involvement of an epigenetic control. The selected lncRNAs were involved in the modulation of lipid metabolism pathway (see Results, module M11), and it is well known that dyslipidemia is frequently associated with PsA (36) and, in particular, patients with PsA have been shown to have elevated cholesterol levels (37) and increase risk of cardiovascular disease.

Another deregulated signaling pathway is the S6K1 (S6 kinase 1) and mTOR (mammalian target of rapamycin) pathway (see Results, modules M1, M4, and M5), a nutrient sensing system that respond to nutrient overload leading to insulin resistance, obesity, and diabetes (38, 39). These observations suggest that lncRNAs may also be involved in the metabolic syndrome frequently associated with PsA. Hyperuricemia is another feature of metabolic syndrome and is also known to be more frequent in psoriatic subjects than in the normal population. In humans, purines are metabolized into uric acid, which is a strong stimulator of innate immunity. In this regard, hyperuricemia may increase uric acid crystallization in and around joints, thereby inducing PsA in psoriatic subjects, thus representing an independent risk factor for PsA. Activation of pathways modulation of genes involved in related to nucleobase, nucleoside, nucleotide, and nucleic acid metabolism (modules M1, M4, and M8) may reflect this particular aspect of PsA.

Finally, lncRNAs control several pathways involved in cell adhesion (see Results, modules M4, M10, M11, M12, M14, and M15). The modulation of these pathways may contribute to the increased mucosal permeability of the gut that has been observed in PsA, even in the absence of bowel pathology (40). Indeed, bowel disease is six times more frequent in patients with PsA. Since an altered intestinal barrier is a feature of PsA, the presence of gastrointestinal involvement in PsA should be carefully investigated during the diagnostic work up.

The detection of significantly different serum levels in PsA patients compared to healthy subjects of several soluble mediators, ascribed to the main pathways targeted by the selected lncRNAs, strongly suggests that they modulate signaling networks that are active in the disease. In particular, the increased serum levels of glypican-4 and the decrease of adiponectin and omentin highlight the typical metabolic aspects of PsA patients.

Indeed glypican-4 has been considered a novel adipokine that boosts insulin signaling and, interestingly glypican-4 levels are significantly increased in obese patients with insulin resistance (41). Moreover, both adiponectin and omentin have been showed decreased in sera from psoriatic patients (42, 43) and decreased levels of both molecules have been associated with, and involved in, metabolic syndrome (44, 45).

In conclusion, this study is the first report on lncRNAs that may exert an epigenetic control on PsA pathogenesis. The original approach that integrates genes and lncRNAs expression profiles presented in the study, allows to identify deregulated lncRNAs that modulate crucial molecular signaling associated with the typical features of the disease. These findings further support that PsA is an autoimmune disease with systemic inflammation associated with obesity and metabolic syndrome leading to cardiovascular disease.

Finally, we may suggest that these lncRNAs may be useful to design novel therapeutic strategies in PsA.

## Ethics Statement

All the participants to the study signed a written informed consent and the local Ethical Committee of the University Hospital of Verona, Verona, Italy, had approved the study protocol. All the investigations have been performed according to the principles of the Helsinki declaration.

## Author Contributions

APU, CL, and MD conceived and designed the experiments. MD, APE, PF, GP, and ET performed the experiments. MD and APE analyzed the data. GP and ET contributed reagents and collected the patients’ samples. MD wrote the paper with input from APU and CL.

## Conflict of Interest Statement

The research was conducted in the absence of any commercial or financial relationships that could be construed as a potential conflict of interest.

## Footnotes

^1^http://www.bioinfo.org/NPInter/ (Accessed: November 16, 2017).

^2^www.funrich.org/ (Accessed: December 15, 2017).

^3^http://string-db.org/ (Accessed: February 06, 2018).

^4^www.cytoscape.org (Accessed: February 15, 2018).

^5^www.geneontology.org (Accessed: January 19, 2018).

## Supplementary Material

Table S1 Long non-coding RNAs modulated in psoriatic arthritis patients versus healthy subjects.

Table S2 Genes modulated in psoriatic arthritis patients versus healthy subjects.

Table S3 Long non-coding RNAs modulated in psoriatic arthritis patients versus healthy subjects with available target annotations.

Table S4 Target genes of the selected microRNAs that are targeted by long non-coding RNAs modulated in psoriatic arthritis.

Table S5 Genes modulated in psoriatic arthritis patients versus healthy subjects and included in the modules.

Table S6 Long non-coding RNAs modulated in psoriatic arthritis that target modules.

Figure S1 Expression of selected long non-coding RNAs in psoriatic arthritis (PsA) patients by real-time PCR. Real-time PCR of LUCAT1 and lnc-TRIM55-1 in PsA and healthy samples included in the microarray. Bars indicate SD. **p* < 0.05; ***p* < 0.01; Student *t*-test.

Figure S2 Expression by real-time PCR of genes involved in signaling pathways that are modulated in psoriatic arthritis patients compared to healthy subjects. Bars indicate SD.

## References

[1] Ritchlin CT Colbert RA Gladman DD Psoriatic arthritis. N Engl J Med (2017) 376:2095–6. 10.1056/NEJMra1505557 28538114

[2] Fitzgerald O Winchester R. Psoriatic arthritis: from pathogenesis to therapy. Arthritis Res Ther (2009) 11:214. 10.1186/ar2580 19232079 PMC2688229

[3] Rahman P Elder JT. Genetic epidemiology of psoriasis and psoriatic arthritis. Ann Rheum Dis (2005) 64(Suppl 2):ii37–9; discussion ii40–1. 10.1136/ard.2004.030775 15708933 PMC1766868

[4] Mease P. Psoriatic arthritis and spondyloarthritis assessment and management update. Curr Opin Rheumatol (2013) 25:287–96. 10.1097/BOR.0b013e32835fd8d5 23492739

[5] Moll JM Wright V Psoriatic arthritis. Semin Arthritis Rheum (1973) 3:55–78. 10.1016/0049-0172(73)90035-8 4581554

[6] Robinson H Kelly S Pitzalis C. Basic synovial biology and immunopathology in psoriatic arthritis. J Rheumatol Suppl (2009) 83:14–6. 10.3899/jrheum.090212 19661529

[7] Costa L Perricone C Chimenti MS Del Puente A Caso P Peluso R Switching between biological treatments in psoriatic arthritis: a review of the evidence. Drugs R D (2017) 17:509–22. 10.1007/s40268-017-0215-7 29058302 PMC5694428

[8] Krueger JG Brunner PM. Interleukin-17 alters the biology of many cell types involved in the genesis of psoriasis, systemic inflammation and associated comorbidities. Exp Dermatol (2018) 27:115–23. 10.1111/exd.13467 29152791

[9] Dolcino M Ottria A Barbieri A Patuzzo G Tinazzi E Argentino G Gene expression profiling in peripheral blood cells and synovial membranes of patients with psoriatic arthritis. PLoS One (2015) 10:e0128262. 10.1371/journal.pone.0128262 26086874 PMC4473102

[10] Pelosi A Lunardi C Fiore PF Tinazzi E Patuzzo G Argentino G MicroRNA espression profiling in psoriatic arthritis. Biomed Res Int (2018) 2018:7305380 10.1155/2018/7305380 29850558 PMC5937573

[11] Wu GC Pan HF Leng RX Wang DG Li XP Li XM Emerging role of long noncoding rnas in autoimmune diseases. Autoimmun Rev (2015) 14:798–805. 10.1016/j.autrev.2015.05.004 25989481

[12] Ahn R Gupta R Lai K Chopra N Arron ST Liao W. Network analysis of psoriasis reveals biological pathways and roles for coding and long non-coding rnas. BMC Genomics (2016) 17:841. 10.1186/s12864-016-3188-y 27793094 PMC5084355

[13] Gupta R Ahn R Lai K Mullins E Debbaneh M Dimon M Landscape of long noncoding rnas in psoriatic and healthy skin. J Invest Dermatol (2016) 136:603–9. 10.1016/j.jid.2015.12.009 27015450 PMC5546103

[14] Wu T Wang J Liu C Zhang Y Shi B Zhu X Npinter: the noncoding rnas and protein related biomacromolecules interaction database. Nucleic Acids Res (2006) 34:D150–2. 10.1093/nar/gkj025 16381834 PMC1347388

[15] Yuan J Wu W Xie C Zhao G Zhao Y Chen R. Npinter v2.0: an updated database of ncrna interactions. Nucleic Acids Res (2014) 42:D104–8. 10.1093/nar/gkt1057 24217916 PMC3965026

[16] Pathan M Keerthikumar S Ang CS Gangoda L Quek CY Williamson NA Funrich: an open access standalone functional enrichment and interaction network analysis tool. Proteomics (2015) 15:2597–601. 10.1002/pmic.201400515 25921073

[17] Jensen LJ Kuhn M Stark M Chaffron S Creevey C Muller J String 8 – a global view on proteins and their functional interactions in 630 organisms. Nucleic Acids Res (2009) 37:D412–6. 10.1093/nar/gkn760 18940858 PMC2686466

[18] Cline MS Smoot M Cerami E Kuchinsky A Landys N Workman C Integration of biological networks and gene expression data using cytoscape. Nat Protoc (2007) 2:2366–82. 10.1038/nprot.2007.324 17947979 PMC3685583

[19] Vriend J Reiter RJ. Melatonin, bone regulation and the ubiquitin-proteasome connection: a review. Life Sci (2016) 145:152–60. 10.1016/j.lfs.2015.12.031 26706287

[20] Takeuchi O. Endonuclease regnase-1/monocyte chemotactic protein-1-induced protein-1 (mcpip1) in controlling immune responses and beyond. Wiley Interdiscip Rev RNA (2018) 9:e1449. 10.1002/wrna.1449 28929622

[21] Chandran V The genetics of psoriasis and psoriatic arthritis. Clin Rev Allergy Immunol (2013) 44:149–56. 10.1007/s12016-012-8303-5 22274791

[22] Sanchez-Tena S Cubillos-Rojas M Schneider T Rosa JL. Functional and pathological relevance of herc family proteins: a decade later. Cell Mol Life Sci (2016) 73:1955–68. 10.1007/s00018-016-2139-8 26801221 PMC11108380

[23] Yeo L Adlard N Biehl M Juarez M Smallie T Snow M Expression of chemokines cxcl4 and cxcl7 by synovial macrophages defines an early stage of rheumatoid arthritis. Ann Rheum Dis (2016) 75:763–71. 10.1136/annrheumdis-2014-206921 25858640 PMC4819606

[24] Lee J Park C Kim HJ Lee YD Lee ZH Song YW Stimulation of osteoclast migration and bone resorption by c-c chemokine ligands 19 and 21. Exp Mol Med (2017) 49:e358. 10.1038/emm.2017.100 28729639 PMC5565950

[25] Messina N Fulford T O’Reilly L Loh WX Motyer JM Ellis D The nf-kappab transcription factor rela is required for the tolerogenic function of foxp3(+) regulatory t cells. J Autoimmun (2016) 70:52–62. 10.1016/j.jaut.2016.03.017 27068879

[26] Yi Z Wallis AM Bishop GA. Roles of traf3 in t cells: many surprises. Cell Cycle (2015) 14:1156–63. 10.1080/15384101.2015.1021524 25723057 PMC4613145

[27] Yao Z Lei W Duan R Li Y Luo L Boyce BF. Rankl cytokine enhances tnf-induced osteoclastogenesis independently of tnf receptor associated factor (traf) 6 by degrading traf3 in osteoclast precursors. J Biol Chem (2017) 292:10169–79. 10.1074/jbc.M116.771816 28438834 PMC5473222

[28] Emslie D D’Costa K Hasbold J Metcalf D Takatsu K Hodgkin PO Oct2 enhances antibody-secreting cell differentiation through regulation of il-5 receptor alpha chain expression on activated b cells. J Exp Med (2008) 205:409–21. 10.1084/jem.20072049 18250192 PMC2271016

[29] Wu B Wang Y Wang C Wang GG Wu J Wan YY. Bptf is essential for t cell homeostasis and function. J Immunol (2016) 197:4325–33. 10.4049/jimmunol.1600642 27799308 PMC5127169

[30] Guggino G Lo Pizzo M Di Liberto D Rizzo A Cipriani P Ruscitti P Interleukin-9 over-expression and t helper 9 polarization in systemic sclerosis patients. Clin Exp Immunol (2017) 190:208–16. 10.1111/cei.13009 28681919 PMC5629425

[31] Koch S Sopel N Finotto S. Th9 and other il-9-producing cells in allergic asthma. Semin Immunopathol (2017) 39:55–68. 10.1007/s00281-016-0601-1 27858144

[32] Karczewski J Dobrowolska A Rychlewska-Hanczewska A Adamski Z. New insights into the role of t cells in pathogenesis of psoriasis and psoriatic arthritis. Autoimmunity (2016) 49:435–50. 10.3109/08916934.2016.1166214 27050731

[33] Bellido T Huening M Raval-Pandya M Manolagas SC Christakos S. Calbindin-d28k is expressed in osteoblastic cells and suppresses their apoptosis by inhibiting caspase-3 activity. J Biol Chem (2000) 275:26328–32. 10.1074/jbc.M003600200 10835428

[34] Weng T Mao F Wang Y Sun Q Li R Yang G Osteoblastic molecular scaffold gab1 is required for maintaining bone homeostasis. J Cell Sci (2010) 123:682–9. 10.1242/jcs.058396 20124419

[35] Pustylnik S Fiorino C Nabavi N Zappitelli T da Silva R Aubin JE Eb1 levels are elevated in ascorbic acid (aa)-stimulated osteoblasts and mediate cell-cell adhesion-induced osteoblast differentiation. J Biol Chem (2013) 288:22096–. 10.1074/jbc.M113.481515 23740245 PMC3724663

[36] Yim KM Armstrong AW. Updates on cardiovascular comorbidities associated with psoriatic diseases: epidemiology and mechanisms. Rheumatol Int (2017) 37:97–105. 10.1007/s00296-016-3487-2 27221457

[37] Wu S Li WQ Han J Sun Q Qureshi AA. Hypercholesterolemia and risk of incident psoriasis and psoriatic arthritis in us women. Arthritis Rheumatol (2014) 66:304–10. 10.1002/art.38227 24504802 PMC4082661

[38] Gulati P Thomas G. Nutrient sensing in the mtor/s6k1 signalling pathway. Biochem Soc Trans (2007) 35:236–8. 10.1042/BST0350236 17371247

[39] Um SH D’Alessio D Thomas G. Nutrient overload, insulin resistance, and ribosomal protein s6 kinase 1, s6k1. Cell Metab (2006) 3:393–402. 10.1016/j.cmet.2006.05.003 16753575

[40] Holden W Orchard T Wordsworth P. Enteropathic arthritis. Rheum Dis Clin North Am (2003) 29:513–530, viii. 10.1016/S0889-857X(03)00043-7 12951865

[41] Zhu HJ Pan H Cui Y Wang XQ Wang LJ Li NS The changes of serum glypican4 in obese patients with different glucose metabolism status. J Clin Endocrinol Metab (2014) 99:E2697–701. 10.1210/jc.2014-2018 25144630

[42] Campanati A Ganzetti G Giuliodori K Marra M Bonfigli A Testa R Serum levels of adipocytokines in psoriasis patients receiving tumor necrosis factor-α inhibitors: results of a retrospective analysis. Int J Dermatol (2015) 54:839–45. 10.1111/ijd.12706 25877149

[43] Zhang C Zhu KJ Liu JL Xu GX Liu W Jiang FX Omentin-1 plasma levels and omentin-1 expression are decreased in psoriatic lesions of psoriasis patients. Arch Dermatol Res (2015) 307:455–9. 10.1007/s00403-015-1549-z 25690163

[44] Frankenberg ADV Reis AF Gerchman F Relationships between adiponectin levels, the metabolic syndrome, and type 2 diabetes: a literature review. Arch Endocrinol Metab (2017) 61:614–22. 10.1590/2359-3997000000316 29412387 PMC10522055

[45] Tan BK Adya R Randeva HS. Omentin: a novel link between inflammation, diabesity, and cardiovascular disease. Trends Cardiovasc Med (2010) 20:143–8. 10.1016/j.tcm.2010.12.002 21742269

