# Supplementary figures and images for: Long Non-Coding RNAs Play a Role in the Pathogenesis of Psoriatic Arthritis by Regulating MicroRNAs and Genes Involved in Inflammation and Metabolic Syndrome
Source: Front Immunol. 2018 Jul 16;9:1533. doi: 10.3389/fimmu.2018.01533 (PMC6054935; doi:10.3389/fimmu.2018.01533)

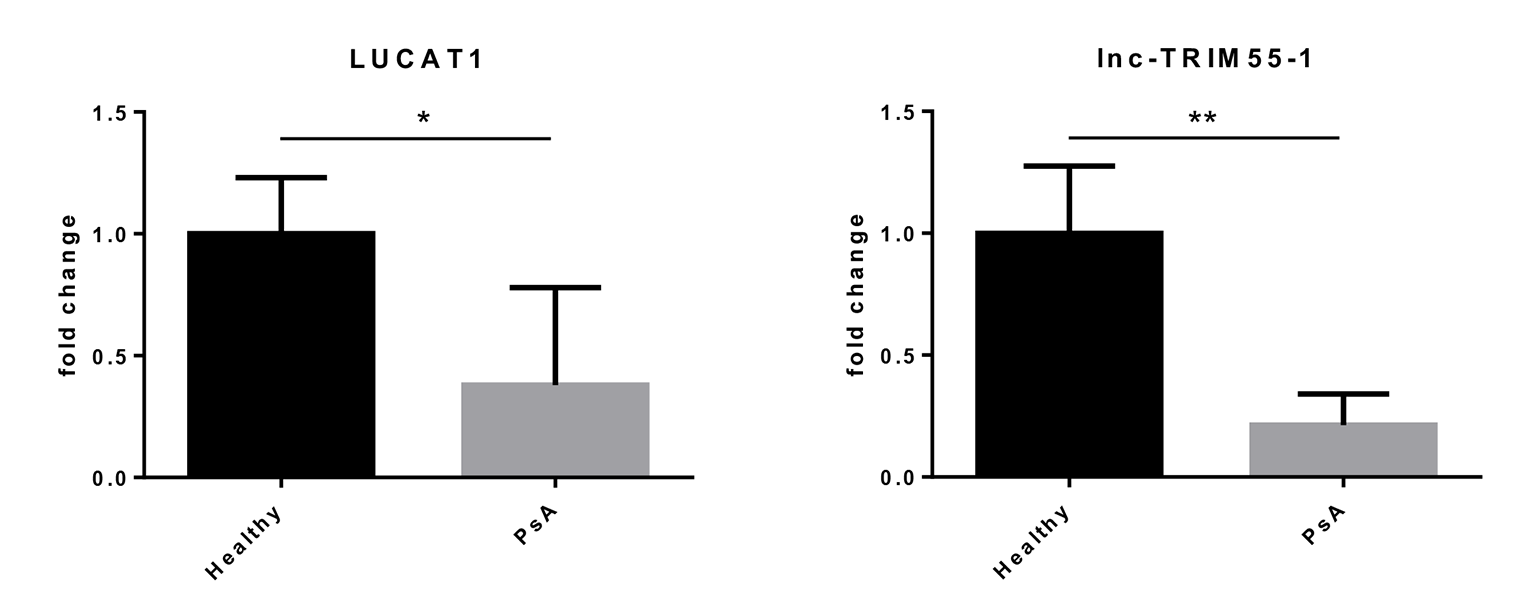

Supplement: Figure S1 — Expression of selected long non-coding RNAs in psoriatic arthritis (PsA) patients by real-time PCR. Real-time PCR of LUCAT1 and lnc-TRIM55-1 in PsA and healthy samples included in the microarray. Bars indicate SD. *p < 0.05; **p < 0.01; Student t-test. [file image_1.tif]

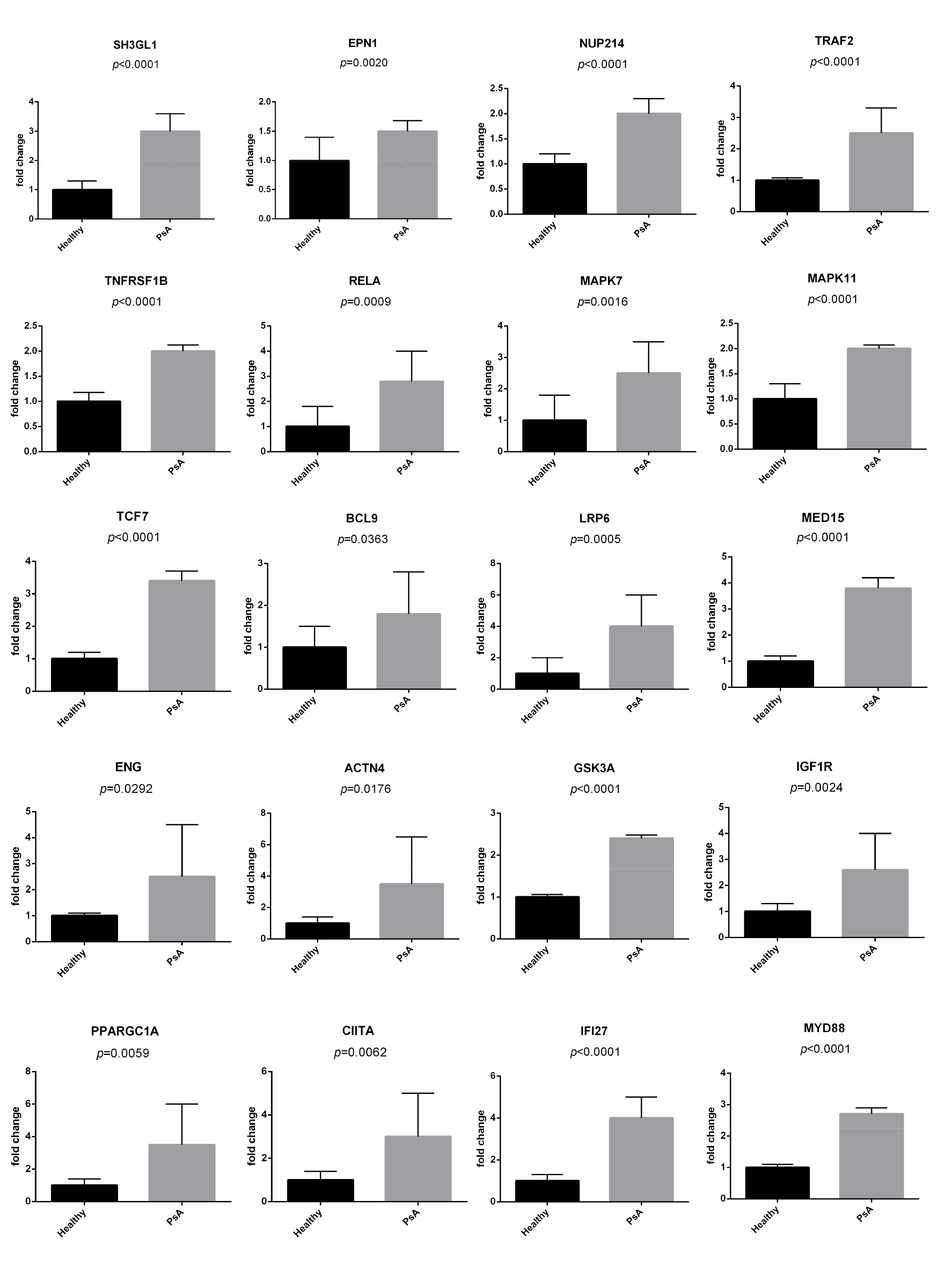

Supplement: Figure S2 — Expression by real-time PCR of genes involved in signaling pathways that are modulated in psoriatic arthritis patients compared to healthy subjects. Bars indicate SD. [file image_2.tif]
